# The Intoxication Effects of Methanol and Formic Acid on Rat Retina Function

**DOI:** 10.1155/2016/4087096

**Published:** 2016-09-04

**Authors:** Dong-Mei Liu, Shu Zhou, Jie-Min Chen, Shu-Ya Peng, Wen-Tao Xia

**Affiliations:** ^1^Shanghai Key Laboratory of Forensic Medicine, Shanghai Forensic Service Platform, Institute of Forensic Sciences, Ministry of Justice, Shanghai 200063, China; ^2^Institute of Shanghai Huayi Forensic Science, Shanghai 200335, China; ^3^Xinguang Institute of Judicial Expertise, Linyi 276017, China

## Abstract

*Objective*. To explore the potential effects of methanol and its metabolite, formic acid, on rat retina function.* Methods*. Sprague-Dawley rats were divided into 3- and 7-day groups and a control. Experimental groups were given methanol and the control group were provided saline by gavage. Retinal function of each group was assessed by electroretinogram. Concentrations of methanol and formic acid were detected by GC/HS and HPLC, respectively.* Results*. The a and b amplitudes of methanol treated groups decreased and latent periods delayed in scotopic and photopic ERG recordings. The summed amplitudes of oscillatory potentials (OPs) of groups B and C decreased and the elapsed time delayed. The amplitudes of OS1, OS3, OS4, and OS5 of group B and OS3, OS4, and OS5 of group C decreased compared with the control group. The IPI1 of group B and IPI1-4 of group C were broader compared with the control group and the IPI1-4 and ET of group B were broader than group C. Conclusions. Both of scotopic and photopic retinal functions were impaired by methanol poisoning, and impairment was more serious in the 7-day than in the 3-day group. OPs, especially later OPs and IPI2, were more sensitive to methanol intoxication than other eletroretinogram subcomponents.

## 1. Introduction

Methanol is a colorless and highly toxic organic solvent and is commonly used in industry. Methanol is rapidly absorbed by oral mucosa and gastrointestinal tract or skin. Subsequently, it is metabolized to formaldehyde by alcohol dehydrogenase, which is in turn converted into formic acid by aldehyde dehydrogenase. It has been reported that formaldehyde stays too transitory to be detected by analytical instruments, while formic acid is easily detected after methanol ingestion [1]. Consequently, formic acid has been considered as the main toxic factor in methanol poisoning [2]. Ocular toxicity is the most significant characteristic in methanol toxic effects and electroretinogram is often used to evaluate the retinal function. Currently, most studies on methanol intoxication focus on a-wave and b-wave subcomponents in photopic and scotopic environment [3, 4]. Our previous research showed that a-wave and b-wave were greatly destroyed upon methanol intoxication [5]. However, very limited researches on the characters of OPs waves upon methanol poisoning were performed. Garner and Lee found that all OPs amplitudes reduced nonspecifically and latencies delayed after acute methanol intoxication [6]. Plaziac et al. reported that OS2 was less affected among all decreased OPs (2–4) waves [7]. Considering that OPs waves are a series of rhythmic low-amplitude potentials superimposed on the ascending phase of b-wave, systemic and deep researches on the characteristic changes of OPs waves will make much sense for exploring the toxic effects of methanol poisoning. In this research, we try to explore the potential effects of methanol and its metabolite, formic acid, on rat retina function though the parameter of OPs waves.

## 2. Materials and Methods

### 2.1. Animal Model for Methanol Intoxication

This research adhered to the tenets of the Declaration of Helsinki or the NIH statement for the Use of Animals in Research.

Thirty adult (200–250 g, National Rodent Laboratory Animal Resources, Shanghai Branch, China) male Sprague-Dawley rats with no ophthalmopathy were supplied with food and water* ad libitum* and maintained at a standard temperature and humid environment. All animal experiments were approved by ethical certification and performed according to the Association for Research in Vision and Ophthalmology statement for the use of animals in ophthalmic and vision research. Rats were maintained in a sealed chamber and given a N_2_O/O_2_ gas mixture (1/1 by vol; flow rate, 2 L/min) for 24 h before administration of methanol or saline and the gas mixture continued until the experiment's end. Initially, animals were randomly classified into control group (A), 3-day group (B), and 7-day group (C) with 10 rats per group. It has been reported that LD_0_ and LD_50_ of oral methanol for rats are 6.67 and 12–14 mL/kg [8]. Groups B and C were treated with methanol by gavage (8 mL/kg per dose, followed by a 4 mL/kg supplemental dose 24 h later), and the control group A was treated with equivalent volumes of saline. All poisoned rats showed a typical toxic condition. ERG recordings of Group B were performed 72 h (3 days) later after methanol treatment and those of group C were performed 168 h (7 days) later. Then, rats from each group were killed by decapitation and cardiac blood samples were collected into ethylenediaminetetraacetic acid anticoagulation tubes.

### 2.2. ERG Recordings

ERG recordings were performed by the Electrophysiological Test Unit (Roland, Germany) as described by ISCEV. Before test, absolutely dark adaptation for 4 h was necessary. Then animals were anesthetized with 10% chloralhydrate (3.5 mL/kg, intraperitoneal injection) and each pupil was dilated by tropicamide compound. After sufficient dilation, the corneal surface was anesthetized with tetracaine hydrochloride. The recording electrode was positioned on the corneal surface, the reference electrode was penetrated into the middle forehead, and the ground electrode was inserted into the skin of the ipsilateral-mastoid process. In the process of scotopic ERG, the flash light intensity of −25 dB was set for recording Rod-response and 0 dB was set for recording Max-response and OPs. Before the photopic ERG recording, the rats' eyes needed 5 min for light adaptation. In photopic ERG process, the flash light intensity of 0 dB was set for recording Cone-response. To diminish the external interference, the amplifier was set to be with voltage of ±1 mv and narrow bandpass between 1 and 300 Hz.

**Figure 1:**
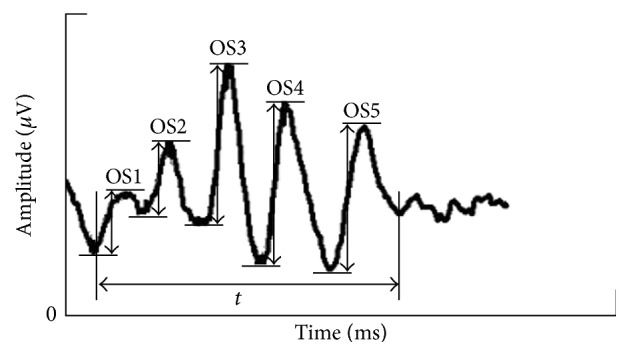
The measurement of amplitudes of OPs response and ET.

Five consecutive wavelets of OPs were carefully measured (Figure 1). Then, the OPs responses were characterized by calculating the individual amplitude of each wave, summed amplitudes of five wavelets of OPs (SAOP), elapsed time (ET) from the appearance of the first wavelet to the end of the fifth wavelet, and interpeak interval (IPI) as the time interval between adjacent peaks of OPs wavelets.

SPSS 20.0 software was used to analyze data with *t*-tests and significant difference (*P*) set at 0.05.

### 2.3. Methanol Concentration from Blood Analysis

Thirty blood samples (ten samples from group A, group B, and group C resp.) were used to determine methanol concentrations by headspace gas chromatography (GC/HS), as previously described [5]. Methanol concentrations were calculated from a standard curve produced using GC/HS results from a series of known concentrations of chromatographic grade methanol.

### 2.4. Formic Acid Concentration from Blood Analysis

The determinations of formic acid concentration were performed on Thermo U3000 HPLC (Thermo Fisher Scientific Inc., Waltham, MA, USA). System parameters included a Calesil ODS-100 C18 chromatographic column (5 *μ*m, 4.6 × 250 mm), ambient temperature, a 1.0 mL/min flow rate, and a methanol/water mixture (6/4 by vol) as the mobile phase. Formic acid was measured with a diode array detector (DAD, Thermo Fisher Scientific Inc.) at 215 nm. Tissues were placed in a tube and shaken in a FastPrep-24 instrument (MP Biomedicals, LLC, Solon, OH, USA) for 1-2 min. Then, 100 *μ*L blood was sufficiently mixed with the mobile phase and centrifuged at 10 000 rpm for 10 min. The 20 *μ*L supernatant was then injected into the high-performance liquid chromatography (HPLC) and formic acid concentrations were determined from a standard curve established from HPLC of standard formic acid solutions.

## 3. Results

### 3.1. Methanol Determination in Blood Using GC/HS

A standard curve formula (*y* = 0.621*x* + 0.112; *R* ^2^ = 0.999) was established to analyze experimental methanol concentrations. The methanol chromatographic peak showed a retention time of 1.143 min. The methanol concentration in blood was 1.00 ± 0.61 mg/mL (*n* = 10) in the 3-day group, while no effective concentration was detected in the 7-day group (Table 1).

### 3.2. Formic Acid Determination Using HPLC

Similarly, a standard curve formula (*y* = 15.837*x* − 6.6471; *R* ^2^ = 0.992) was established to analyze experimental formic acid concentrations. The standard formic acid chromatographic peak showed a retention time of 1.977–2.07 min. Blood formic acid concentrations of 0.61 ± 0.07 mg/mL and 0.60 ± 0.05 mg/mL were found in the 3- and 7-day groups, respectively (Table 1).

**Figure 2:**
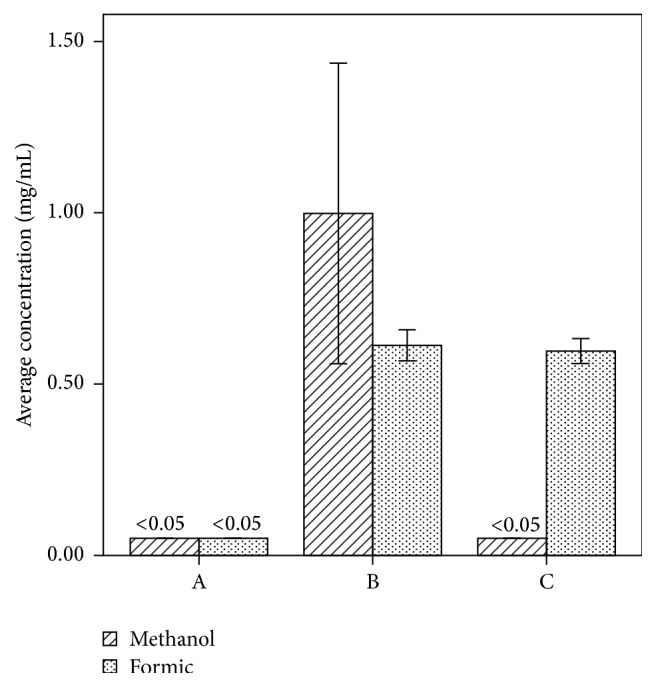
Methanol and formic acid concentrations in blood. Compared with methanol, the formic acid metabolite was significantly accumulated in blood.

Compared with methanol, its metabolite, formic acid, persistently accumulated in blood in methanol-treated groups (Figure 2).

### 3.3. ERG Recordings

For Rod-response of ERG, the a and b amplitudes of group B decreased approximately to 53% and 37%, respectively, and b amplitude of group C decreased approximately to 48% compared with the control group (*P* < 0.05, the exact *P* value in Table 2).

For Max-response of ERG, the a and b amplitudes of group B decreased approximately to 53% and 40% and b amplitude of group C decreased approximately to 44% compared with the control group (*P* < 0.05, the exact *P* value in Table 3). The latent periods of a wavelet of group B were delayed approximately to 26% in Max-response, and latent period of a wavelet of group C was delayed to 28% compared with the control group (*P* < 0.05, the exact *P* value in Table 3).

**Figure 3:**
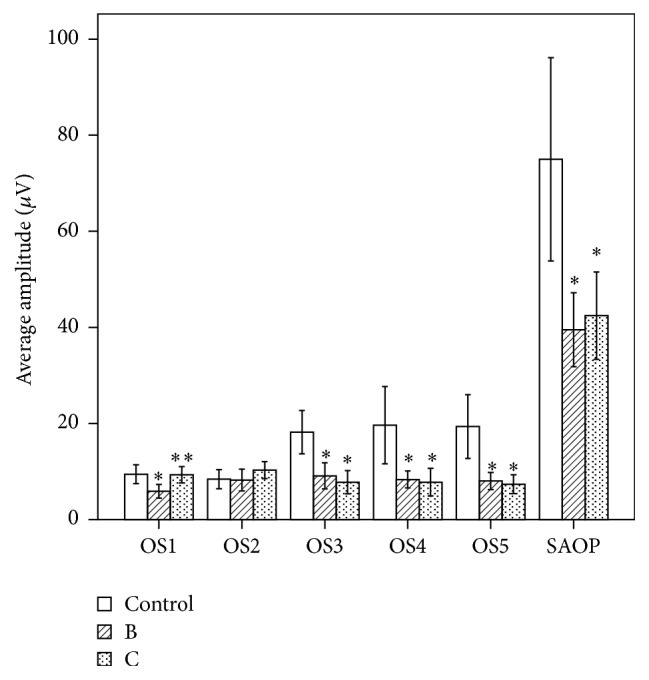
Comparison of individual amplitude and SAOP between three groups (*n* = 20). Amplitudes of OS1, OS3, OS4, OS5, and SAOP of group B and OS3, OS4, OS5, and SAOP of group C were significantly lower than the control group (^*∗*^ *P* < 0.05). Comparison of OS1 value between 3- and 7-day groups was statistically significant (^*∗∗*^ *P* < 0.05).

**Figure 4:**
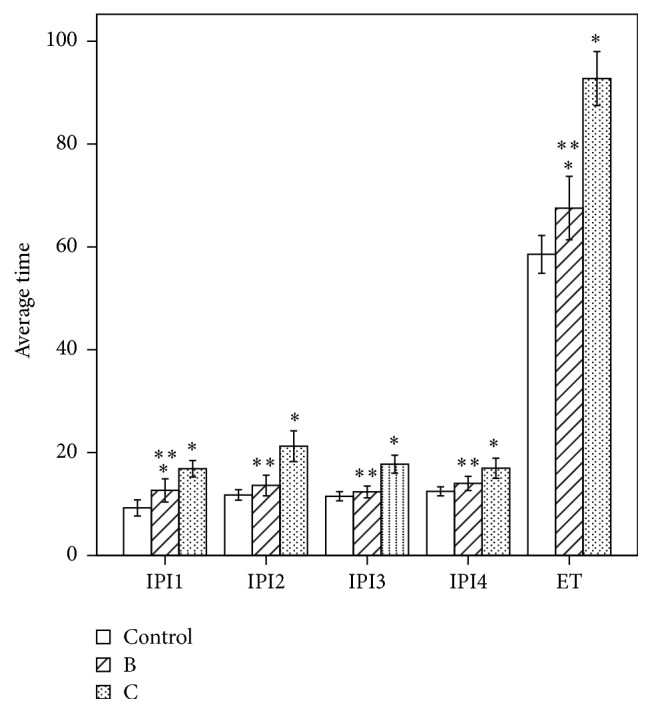
Comparison of IPI and ET between the three groups shown above (Figure 2, *n* = 20). Compared with the control group, IPI1 and ET of group B and IPI1-4 and ET of group C were longer than the control group (^*∗*^ *P* < 0.05). The IPI1-4 and ET of group B were longer than group C (^*∗∗*^ *P* < 0.05).

The summed amplitudes of oscillation potentials of groups B and C decreased to approximately 53% and 57% compared with the control (*P* < 0.05, the exact *P* value in Table 4) and the elapsed time was delayed to approximately 15% and 60% (*P* < 0.05, the exact *P* value in Table 4). The amplitudes of OS1, OS3, OS4, and OS5 of group B and OS3, OS4, and OS5 of group C decreased obviously compared with the control group (*P* < 0.05). Comparison of OS1 value between B and C groups was statistically significant (*P* < 0.05). The IPI1 of group B and IPI1-4 of group C were broader compared with the control group (*P* < 0.05) and the IPI1-4 and ET of group B were broader than group C (*P* < 0.05) (Figures 3 and 4).

For Cone-response, the a and b amplitudes of group B decreased approximately to 53% and 40% and b amplitude of group C decreased approximately to 69% compared with the control group (*P* < 0.05, the exact *P* value in Table 5). Comparison of b amplitudes between B and C groups was statistically significant (*P* < 0.05, the exact *P* value in Table 5). The latent periods for a-wave and b-wave of group C were delayed approximately to 29% and 9% compared with the control group (*P* < 0.05, the exact *P* value in Table 5).

## 4. Discussion

In recent years, methanol poisoning events occurred frequently because of adulterated wine and occupational exposure. Methanol poisoning severely damages the retina and optic nerve resulting in the impairment of vision and visual field [9]. In addition to methanol, its metabolites, formic acid and formaldehyde, have also been attributed as neurotoxic induced by methanol poisoning and might be more toxic than methanol itself [10]. The severity of methanol toxicity correlates with accumulation of methanol and its metabolite, formic acid, in blood after methanol administration [6].

The successful detection of toxicant is extremely important for diagnosis and treatment of intoxication. Chromatographic methods, including GC/HS and HPLC, are powerful tools in analytical chemistry. GC/HS is widely used to detect volatile compounds, such as ethanol and methanol [11]. Our laboratory had successfully detected methanol concentrations from methanol poisoning serum [5]. Although some studies have simultaneously analyzed methanol and formic acid concentrations in postmortem samples using GC/HS, unfortunately, this method was not widely recognized as an effective method to detect formic acid concentration in blood or tissues [12]. Morris successfully quantified formic acid concentrations by HPLC at 210 nm absorbance, which was used in the present study to detect and analyze formic acid concentrations after methanol intoxication [13].

In our present study, although methanol in blood was nearly completely metabolized after 7 days of intoxication, quantified and closed formic acid concentrations were detected in blood in 3- and 7-day intoxication groups. That is to say, formic acid as the continuation of methanol toxicity has a persistently cumulative phenomenon in blood comparing with methanol. It is likely that methanol and its metabolite, formic acid, commonly contributed to the toxic effects of methanol intoxication on retina structure and function and the effect of formic acid might be more persistent.

ERG technique is widely used to assess retinal function. International Society for Clinical Electrophysiology of Vision (ISCEV) Standard for full-field clinical electroretinography (2008 update) specified five responses including Dark-adapted 0.01 ERG (Rod-response), Dark-adapted 3.0 ERG (Max-response), Dark-adapted 3.0 oscillatory potentials (OPs), Light-adapted 3.0 ERG (Cone-response), and Light-adapted 3.0 flicker (30 Hz flicker) [14], the first four responses of which were recorded in this study except 30 Hz flicker. The results showed that a and b amplitudes of methanol intoxication groups were decreased and some of the latent periods of a- and b-waves were delayed. Methanol intoxication destroyed both of the scotopic and photopic ERG recordings, which were in accordance with the previous research of our laboratory [5].

As a scotopic response of ERG recordings, OPs waves separated from b-wave through fourier spectrum filtering technology can be classified into early, intermediate, and late subgroups [15]. Neurosensory retinal cells, including bipolar, amacrine, and interplexiform cells, are directly or indirectly involved in OPs generation and different synaptic activities might excite different oscillatory peaks [16]. The abnormal scotopic OPs were found in some after cisplatin toxicity [17]. OP amplitudes measured in mercury vapor exposure patients were smaller than those of controls for O2 and O3 [18].

In this present study, we found that OPs responses were influenced after methanol intoxication. The reductions of SAOP and delays of ET were confirmed in both two methanol-treated groups indicating the damage of neurosensory retinal cell function. The ET of OPs in 7-day intoxication group was more delayed than in the 3-day group, which was not found in other ERG responses. Most of the comparisons of amplitude and latent periods of both acotopic and photopic ERG recordings between 3-day and 7-day groups methanol-treated groups have no remarkable significance, the possible cause of which may be that damage of methanol poisoning was persistent on both acotopic and photopic ERG recordings. Although the methanol was completely metabolized, its metabolite, formic acid, was continuously accumulated in the 7-day group, which might impede the recovery of retinal dysfunction. The other possible reason is that acotopic and photopic ERG recordings reflect the whole retinal function, are less sensitive than OPs, and cannot respond to the subtle changes of retinal function. The severe delay of ET in later methanol-treated rats indicated that OPs might be more sensitive to methanol poisoning. It is found that the amplitudes of later OPs wavelets in methanol-treated groups, such as OS3, OS4, and OS5, were clearly decreased compared with controls. This indicated that later OPs wavelets were more vulnerable to damage after methanol intoxication, which was consistent with reports by Plaziac et al. [7] but different from Garner and Lee [6]. The reason for this was possibly due to a different method to measure OPs wavelets by Garner and Lee. Previous research, focused on OPs chemical sensitivity, has suggested that later OPs are more sensitive to ethanol than earlier ones [19], which could be an anastomotic explanation for our results. Compared with the control group, the observed longer IPI1, IPI2, IPI3, and IPI4 were more serious in the 7-day than the 3-day group. Moreover, IPI2, which stands for the interval between adjacent peaks of OS2 and OS3, was found to be longer than the other analyzed IPIs, indicating that IPI2 might have been more sensitive to retinal functional changes after methanol poisoning.

Combined with the methanol and formic acid concentrations in blood, deterioration of retinal function in the 7-day group was more possibly due to formic acid accumulation rather than methanol itself. This present study proposed that methanol and formic acid commonly contributed to retinal dysfunction and formic acid might play a major role in the process.

## 5. Conclusions

The present results indicated that both of scotopic and photopic retinal functions were impaired by methanol poisoning and impairment was more serious in the 7-day than in the 3-day group, the reason for which might have been formic acid accumulation in blood after methanol ingestion. Compared with other electroretinogram subcomponents, OPs, especially later OPs and IPI2 were more sensitive to methanol intoxication.

## Figures and Tables

**Table 1:** Methanol and its metabolite formic acid concentrations in blood $\left(\overset{-}{x}\pm s\right)$.

Group	Methanol concentrations in blood (mg/mL)	Formic acid concentrations in blood (mg/mL)
Control (A)	<0.05	<0.05
3-day group (B)	1.00 ± 0.61	0.61 ± 0.07
7-day group (C)	<0.05	0.60 ± 0.05

*n* = 10.

**Table 2:** The latent period and amplitude of Rod-response $\left(\overset{-}{x}\pm s\right)$.

Group	a-wave		b-wave	
	Latent period (ms)	Amplitude (*µ*v)	Latent period (ms)	Amplitude (*µ*v)
A	20.59 ± 5.78	18.59 ± 12.33	68.59 ± 15.80	67.26 ± 29.08
B	24.44 ± 12.33	9.80 ± 9.43^(a)^	58.89 ± 16.91	25.21 ± 14.35^(b)^
C	27.79 ± 18.49	15.48 ± 14.80	59.21 ± 23.89	32.39 ± 16.39^(c)^

*n* = 20 (eye). ^(a)^ *P* = 0.021, ^(b)^ *P* = 0.000, and ^(c)^ *P* = 0.000, and the related latent period and amplitude of intoxication groups were worse than the control group.

**Table 3:** The latent period and amplitude of Max-response $\left(\overset{-}{x}\pm s\right)$.

Group	a-wave		b-wave	
	Latent period (ms)	Amplitude (*µ*v)	Latent period (ms)	Amplitude (*µ*v)
A	16.10 ± 5.46	53.00 ± 27.27	48.45 ± 8.28	163.78 ± 64.18
B	20.35 ± 3.79^(a)^	28.19 ± 14.03^(c)^	43.4 ± 7.02	65.96 ± 21.02^(d)^
C	20.6 ± 5.5^(b)^	39.83 ± 28.72	46.75 ± 4.92	72.84 ± 24.57^(e)^

*n* = 20 (eye). ^(a)^ *P* = 0.007, ^(b)^ *P* = 0.013, ^(c)^ *P* = 0.001, ^(d)^ *P* = 0.000, and ^(e)^ *P* = 0.000, and the related latent period and amplitude of intoxication groups were worse than the control group.

**Table 4:** The SAOP and ET of OPs responses $\left(\overset{-}{x}\pm s\right)$.

Group	A	B	C
SAOP (*µ*v)	74.99 ± 45.25	39.50 ± 16.44^(a)^	42.45 ± 19.44^(b)^
ET (ms)	58.55 ± 7.86	67.55 ± 13.18^(c)^	92.75 ± 11.22^(d)(1)^

*n* = 20 (eye). ^(a)^ *P* = 0.002, ^(b)^ *P* = 0.005, ^(c)^ *P* = 0.013, and ^(d)^ *P* = 0.000, and the related latent period and amplitude of intoxication groups were worse than the control group. ^(1)^ *P* = 0.000, and the related latent period and amplitude of group C were worse than group B.

**Table 5:** The latent period and amplitude of Cone-response $\left(\overset{-}{x}\pm s\right)$.

Group	a-wave		b-wave	
	Latent period (ms)	Amplitude (*µ*v)	Latent period (ms)	Amplitude (*µ*v)
A	17.16 ± 4.37	17.77 ± 9.80	43.16 ± 3.50	79.76 ± 18.94
B	20.20 ± 5.13	9.40 ± 10.56^(b)^	43.80 ± 5.61	31.91 ± 17.37^(d)^
C	22.20 ± 8.14^(a)^	16.13 ± 14.04	47.05 ± 6.22^(c)^	55.00 ± 26.99^(e)(1)^

*n* = 20 (eye). ^(a)^ *P* = 0.022, ^(b)^ *P* = 0.015, ^(c)^ *P* = 0.022, ^(d)^ *P* = 0.000, and ^(e)^ *P* = 0.002, and the related latent period and amplitude of intoxication groups were worse than the control group. ^(1)^ *P* = 0.003, and the related latent period and amplitude of group C were worse than group B.
